# Higher number of *Helicobacter pylori *CagA EPIYA C phosphorylation sites increases the risk of gastric cancer, but not duodenal ulcer

**DOI:** 10.1186/1471-2180-11-61

**Published:** 2011-03-24

**Authors:** Sérgio A Batista, Gifone A Rocha, Andreia MC Rocha, Ivan EB Saraiva, Mônica MDA Cabral, Rodrigo C Oliveira, Dulciene MM Queiroz

**Affiliations:** 1 Laboratory of Research in Bacteriology, Faculdade de Medicina, Universidade Federal de Minas Gerais. Av. Alfredo Balena, 190, sala 216. Belo Horizonte, Minas Gerais, 30130-100, Brazil; 2 Departament of Pathology, Faculdade de Medicina, Universidade Federal de Minas Gerais, Belo Horizonte, Brazil; 3 Laboratory of Cellular and Molecular Immunology, CPqRR-FIOCRUZ, Belo Horizonte, Brazil

## Abstract

**Background:**

*Helicobacter pylori *infection is one of the most common infections worldwide and is associated with gastric cancer and peptic ulcer. Bacterial virulence factors such as CagA have been shown to increase the risk of both diseases. Studies have suggested a causal role for CagA EPIYA polymorphisms in gastric carcinogenesis, and it has been shown to be geographically diverse. We studied associations between *H. pylori *CagA EPIYA patterns and gastric cancer and duodenal ulcer, in an ethnically admixed Western population from Brazil. CagA EPIYA was determined by PCR and confirmed by sequencing. A total of 436 patients were included, being 188 with gastric cancer, 112 with duodenal ulcer and 136 with gastritis.

**Results:**

The number of EPIYA C segments was significantly associated with the increased risk of gastric carcinoma (OR = 3.08, 95% CI = 1.74 to 5.45, p < 10^-3^) even after adjustment for age and gender. Higher number of EPIYA C segments was also associated with gastric atrophy (p = 0.04) and intestinal metaplasia (p = 0.007). Furthermore, patients infected by *cag*A strains possessing more than one EPIYA C segment showed decreased serum levels of pepsinogen I in comparison with those infected by strains containing one or less EPIYA C repeat. Otherwise, the number of EPIYA C segments did not associate with duodenal ulcer.

**Conclusions:**

Our results demonstrate that infection with *H. pylori *strains harbouring more than one CagA EPIYA C motif was clearly associated with gastric cancer, but not with duodenal ulcer.

Higher number of EPIYA C segments was also associated with gastric precancerous lesions as demonstrated by histological gastric atrophic and metaplastic changes and decreased serum levels of pepsinogen I.

## Background

*Helicobacter pylori *colonizes the stomach of more than half of the world's population and is associated with development of complications such as peptic ulcer disease, gastric cancer, and gastric mucosa-associated lymphoid tissue lymphoma [1-4]. The factors that lead few individuals to develop the associated diseases, while the majority of infected people remain asymptomatic, are unknown, but they have been subject of intense research. Among the host factors, cytokine gene polymorphisms were shown to increase the risk of gastric cancer, specifically *IL1B*-31, *IL1RN*, and *TNFA*-307 single nucleotide polymorphisms in European populations, and *IL1RN *in a Brazilian population [5-9]. Pathogen strain-specific factors have been strongly investigated. Among them, the CagA protein is accepted as a risk factor for both peptic ulcer disease and gastric cancer [5,10-12]. In a study of our group, infection by *H. pylori cag*A-positive strains had an odds ratio (OR) of 11.9 for gastric cancer, after adjusting for host polymorphisms and other variables, whereas the strongest host factor was *IL1RN *2 allele, with an OR of 1.9 [5].

*cag*A belongs to a *cag *PAI (pathogenicity island) that codes a type IV secretion system (T4SS) associated with increased secretion of IL-8, a very strong proinflammatory chemokine that participates in the gastritis induced by *H. pylori *infection. The T4SS is also responsible for the entrance of CagA protein into the gastric epithelial cells where CagA is phosphorylated on the tyrosine residue within the phosphorylation motifs in the carboxi-terminal variable region of the protein. These motifs are defined as EPIYA (Glu-Pro-Ile-Tyr-Ala) A, B, C and D according to different flanking aminoacids. CagA protein nearly always possesses EPIYA A and B segments that are followed by none, one, two or three C segments, in strains circulating in the Western countries, or a D segment, in East Asian countries. The EPIYA C and D are the main sites for phosphorylation of CagA. Phosphorylated CagA forms a physical complex with SHP-2 phosphatase and triggers abnormal cellular signals leading to deregulation of cell growth, cell to cell contact and cell migration, elongation of epithelial cells and increase of epithelial cell turnover, which enhance the risk of damaged cells to acquire precancerous genetic changes. Carrying the type D EPIYA or multiple C repeats is associated with increased SHP-2 phosphatase activity induced by CagA [13,14], which raises the possibility that infection by CagA strains possessing higher number EPIYA C segments predisposes to precancerous lesions and gastric cancer.

In fact, this hypothesis has been tested in Eastern countries, but the study results are discordant. Azuma *et al*. [15] found increased proportion of EPIYA D strains among patients with atrophic gastritis and gastric cancer, but other authors have been unable to reproduce these results [16,17]. Similarly, in Western populations, significant association between gastric cancer and increased number of EPIYA C motifs could be demonstrated in two studies [18,19], maybe either by the small number of included patients in the other studies [20-22], or by regional/ethnics differences as already demonstrated for other *H. pylori *virulence markers [23,24]. Furthermore, discrepancies have been also demonstrated in studies evaluating the number of EPIYA C motifs and duodenal ulcer [19,25], which deserves in deep investigations because duodenal ulcer and gastric cancer are mutually exclusive *H. pylori*-associated diseases.

Therefore we evaluated whether increased number of CagA EPIYA C phosphorylation motifs is associated with gastric cancer and/or duodenal ulcer including a large series of patients to avoid bias and to allow adjustment for age and gender in a Western population from Brazil. Since duodenal ulcer and gastric carcinoma are mutually exclusive diseases, and *cag*A is a risk factor for both conditions, we also evaluated whether the number of EPIYA C segments of the strains isolated from patients with duodenal ulcer differed from that of the strains isolated from gastric cancer patients. Because gastric atrophic and metaplastic changes - precancerous lesions - lead to impairment of the production of pepsinogen I (PGI) by chief and mucous neck cells in the corpus and fundic glands, we evaluated whether the higher number of EPIYA C motifs was associated with the serum pepsinogen levels.

## Results

The characteristics of the patients are shown in the Table 1. The presence of *H. pylori*-specific *ure*A and *16S rRNA *was successfully confirmed by PCR in all studied strains and the *cag*A PCRs were positive, by at least one of the method used, in all strains.

**Table 1:** Patient characteristics and distribution of CagA EPIYA genotypes according to *H. pylori*-associated diseases

	**Gastritis 136 (%)**	**Gastric cancer 188 (%)**	**Duodenal ulcer 112 (%)**
	
Mean Age (SD)	52.5 (16.9)	62.3 (13.9)	43.5 (15.1)
Male sex	48 (35.3)	114 (60.6)	53 (47.3)
EPIYA-AB	3 (2.2)	3 (1.6)	4 (3.6)
EPIYA-ABC	108 (79.4)	107 (56.9)	93 (83.0)
EPIYA-ABCC	21 (15.4)	65 (34.6)	15 (13.4)
EPIYA-ABCCC	4 (3.0)	13 (6.9)	0 (0.0)

SD, Standard Deviation

### Determination of the CagA EPIYA pattern

**Figure 1:**
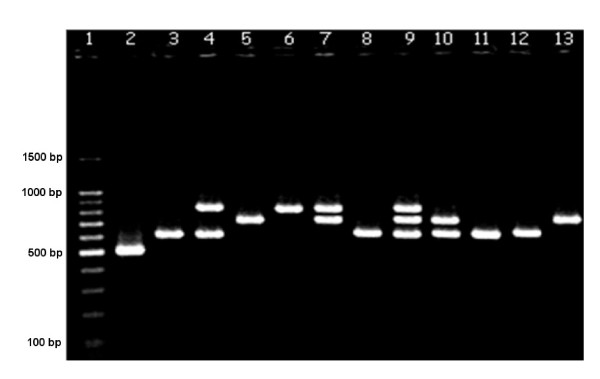
**Electrophoresis of representative samples with each of the CagA EPIYA types seen in patients with *H. pylori*-associated diseases**. Column 1: 100 bp standard; Column 2: EPIYA-AB; Columns 3, 8, 11, and 12: EPIYA-ABC; Column 4: EPIYA-ABC + -ABCCC; Columns 5 and 13: EPIYA-ABCC; Column 6: EPIYA ABCCC; Column 7: EPIYA-ABCC + -ABCCC; Column 9: EPIYA-ABC + -ABCC + -ABCCC; Column 10: EPIYA-ABC + -ABCC.

PCR amplified products from all *cag*A-positive strains showed distinct patterns in the 3' variable region of *cag*A. An electrophoresis gel representing the different CagA EPIYA types is shown in the Figure 1. The PCR results were confirmed by sequencing in seventy five randomly selected PCR products from patients of each group.

No EPIYA D was found in the *H. pylori *strains studied. The distribution of the EPIYA genotypes is shown in the Table 1.

### Association between the numbers of EPIYA C segments and gastric cancer and duodenal ulcer

Colonization by *H. pylori *CagA-positive strains possessing two or three EPIYA C motifs was more frequently observed (p < 10^-3^) in the gastric cancer (78/188, 41.5%) than in the gastritis (25/136, 18.4%) patients. The association remained strongly significant even after adjusting for age and gender by means of logistic regression (Table 2). The Hosmer-Lemeshow test showed good fitness of the model (Chi-square = 3.98, 8 degrees of freedom, p = 0.86, with 10 steps). Otherwise, the number of EPIYA C segments did not associate with duodenal ulcer (Table 2).

**Table 2:** Association between number of CagA EPIYA C motifs and *H. pylori*-associated diseases

	Univariate analysis	Multivariate analysis		
	
	*p*	OR	95% CI	*p*
Gastric cancer				
- Increasing age	< 10^-3^	1.04	1.03 - 1.06	< 10^-3^
- Female sex	< 10^-3^	0.29	0.18 - 0.48	< 10^-3^
- High-risk EPIYA (ABCC or ABCCC)	< 10^-3^	3.08	1.74 - 5.45	< 10^-3^
Duodenal ulcer				
- Increasing age	< 10^-3^	1.03	1.02 - 1.05	< 10^-3^
- Female sex	0.04	1.26	0.73 - 2.18	0.41
- High-risk EPIYA (ABCC or ABCCC)	0.29	-	-	-

The Hosmer-Lemeshow test showed good fitness of the model of gastric cancer (8 degrees of freedom, p = 0.86, with 10 steps) and duodenal ulcer (8 degrees of freedom, p = 0.25, with 10 steps).

Because it might be speculated that the number of EPIYA C motifs increases with increasing age, we also constructed a model with the number of EPIYA C being the dependent variable and the age, sex and *H. pylori*-associated diseases as independent covariables. Increased EPIYA C segments did not associate with age (p = 0.13), sex (p = 0.66) and duodenal ulcer (p = 0.29) but remained associated with gastric cancer (p < 10^-3^, OR = 2.81; 95% CI = 1.64 - 4.82).

### Association between mixed strain colonization and diseases

Mixed strain infection was observed in 57 (13.08%) patients and it was significantly more frequent in patients with gastric cancer (38/188, 20.2%) than in those with gastritis (14/136, 10.3%) with an OR for gastric carcinoma of 2.21 (95%CI = 1.10 to 4.50). Otherwise, mixed infection was less frequently observed in duodenal ulcer patients (5/112, 4.5%) with a trend to a negative association (p = 0.09).

### Association between the numbers of EPIYA C segments and serum PGI levels

The pepsinogen I serum levels were significantly higher (p = 0.01) in duodenal ulcer (mean 161.67 ± 102.36 μg/L) than in gastritis (100.37 ± 70.85 μg/L).

The patients infected by CagA strains possessing two or three EPIYA C segments showed decreased levels of PGI when compared with those with infection by CagA strains possessing ≤ 1 EPIYA C segment (duodenal ulcer: 179.67 ± 83.30 vs. 67.01 ± 34.30, respectively, p = 0.02 and gastritis: 109.26 ± 85.61 vs. 57.55 ± 34.61, respectively, p = 0.01).

### Association between the numbers of EPIYA C repeats and gastric histological alterations and tumour classification

Increased number of EPIYA C segments was associated with the presence of precancerous lesions, either atrophy (p = 0.04) or intestinal metaplasia (p = 0.007), but not with the other histological parameters. Also, the infection by strains carrying increased EPIYA C motifs did not associate with intestinal or diffuse tumour type (p = 0.34).

## Discussion

In this study, by evaluating a large series of patient, we demonstrated that those infected by CagA-positive *H. pylori *strains possessing more than one EPIYA C motif are at thrice-fold increased risk for developing gastric cancer. The strengths of our study include adequate sample size, identification of strains by means of PCR with confirmation by sequencing and adjustment for confounding factors by logistic regression analysis.

CagA is considered to be an important bacterial virulence factor associated with both gastric adenocarcinoma and duodenal ulcer disease [2,5,11,12,26]. The number and pattern of phosphorylation motifs seem to further stratify the risk associated with individual strains [18,27].

It has been demonstrated that *H. pylori *CagA EPIYA patterns have a significant geographic variability and closely follow patterns of historical human migrations. EPIYA D is a characteristic Asian EPIYA pattern that virtually does not occur in the Western *H. pylori *strains [28]. The Brazilians form an unique Western population because, despite the multiple origins and the consequent wide diversity of phenotypic appearance, there has been a substantial degree of inter-ethnic breeding and thus most individuals cannot be ascribed to any of the founding groups on the basis of genetic background, rather they carry about 33% of genes from each of the major races that historically composed the country (Caucasians, Africans and Amerindians) [29]. With this background, it would be expected to find some CagA EPIYA D in our *H. pylori *strains, as it has been detected among Amerindians (in keeping with the theory that initially people from Asia populated the Americas migrating from the East Asia), but we did not detect any EPIYA D in our population.

Unfortunately, there are few studies in respect to the association between EPIYA C number and *H. pylori *associated diseases in Western populations with discordant results among them. Basso *et al*. [19] showed that higher number of EPIYA C segments was associated with gastric carcinoma in a Caucasian population from Italy, similarly to the results of Yamaoka *et al*. [18] evaluating American patients from Texas. Otherwise, no association was observed when Colombian patients were evaluated in the Yamaoka's study [18] in accordance with the results obtained by Acosta *et al*. [22], whereas Sicinschi *et al*. [21] observed associations between increased EPIYA C segments and precancerous lesions. Also, non-conclusive results published by Argent *et al*. [20] evaluating 44 strains from African patients the authors showed tendency of association between CagA with two or more EPIYA C segments and gastric cancer.

These differences may be explained by different study designs, sample size, populations and geographical diversity of *H. pylori *markers of pathogenicity, in respect to the CagA EPIYA pattern, highlighting the need of studying different geographical regions.

The results of this study showed that higher number of EPIYA C segments is associated with gastric cancer and with pre-malignant lesions, atrophy and intestinal metaplasia of the corpus mucosa in the group of patients with gastritis. In agreement with these findings, we also demonstrated that serum concentration of PGI was twice decreased in the patients infected by *cag*A-positive strains with two or three EPIYA C motifs. Because PGI is secreted by chief cells and mucous neck cells in the corpus and fundic glands, serum PGI levels reflect the functional and morphologic status of the oxyntic gastric mucosa; thus, gastric atrophic/metaplastic changes lead to decreased PGI serum levels [30,31]. Although the corpus mucosa of patients with *H. pylori *associated duodenal ulcer is either mildly or not inflamed, the PGI serum levels were also decreased in duodenal ulcer patients infected by strains containing higher number of EPIYA C segments.

The results of the present study strengthen the potential role of CagA polymorphism in the development of gastric cancer in agreement with the results of the previous studies [18,19]. However, we can not exclude the possibility that the genetic constitution of the host, more than the bacterium strain, might predispose to atrophic gastritis and the *H. pylori *strains carrying increasing numbers of EPIYA C repeats would have an advantage over other strains in colonizing the new gastric environment or alternatively a more complex interplay of both mechanisms.

In respect to duodenal ulcer, also the results of the studies are discordant [19,25]. Our results are in agreement with those reported by Basso *et al*. [19] who also did not find association between the disease and the number of EPIYA C segments in an Italian population. Notably, none patient with duodenal ulcer of our cohort was colonized by CagA possessing three EPIYA C segments. As suggested by Yamaoka *et al*. [18], it is possible that strains with higher number of EPIYA C segments may be less resistant to the acid [18].

We also evaluated whether colonization by different strains (mixed infection) could be associated with disease outcomes. We found that gastric cancer patients were significantly more often colonized by mixed strains, whereas patients with duodenal ulcer had a trend toward less mixed strain colonization. One possibility is that patients with gastric cancer would have areas of gastric mucosa showing cancer transformation, alternating with areas of atrophy, intestinal metaplasia, dysplasia, and normal mucosa, each of them representing microenvironments that would be selectively advantageous to mixed infections [32,33].

## Conclusions

In conclusion, we found that infection by *H. pylori *CagA-positive strains harbouring multiple EPIYA C repeats is associated with gastric precancerous lesions and gastric cancer, but not with duodenal ulcer in an ethnically diverse, admixed, Western population.

Although infection by *H. pylori cag*A-positive strains is a risk factor for the mutually exclusive diseases, gastric cancer and duodenal ulcer, CagA strains possessing higher number of EPIYA C segments were associated with gastric cancer, but not with duodenal ulcer.

Higher number of EPIYA C segments was also associated with gastric precancerous lesions as demonstrated by histological gastric atrophic and metaplastic changes and decreased serum levels of pepsinogen I.

## Methods

### Patients

We included 436 patients infected with *cag*A-positive strains of *H. pylori *(188 with gastric cancer, 112 with duodenal ulcer and 136 with gastritis), among those who were submitted to endoscopy to clarify the origin of symptoms related to the upper gastrointestinal tract or who underwent gastric surgery to remove gastric carcinoma at the University Hospital/UFMG, Luxemburgo, and Mário Penna Hospitals, in Belo Horizonte, Brazil. Most of the included individuals (>80%) were of low socioeconomic level with similar cultural habits, and all were native of Minas Gerais state with the same ethnic background, approximately 33% of Portuguese, 33% of Amerindian and 33% of African ancestry, homogeneously present in each subject [29].

The study was approved by the institutional Ethics Committees and informed consent was obtained from all patients. The transport, culture, and microbiological identification of the bacterial isolates were performed as previously described [34,35].

### Histology

In the group of gastritis and duodenal ulcer patients, endoscopic biopsy samples of the antral and oxyntic gastric mucosa were obtained for histological and microbiological study. Antral and oxyntic biopsy specimens were fixed in 10% formalin and embedded in paraffin wax, and 4-μm-thick histological sections were stained with carbolfuchsin for *H. pylori *investigation [35] and hematoxycilin and eosin for histological evaluation according to the updated Sydney System [36]. In the group of gastric cancer patients, the fragments were obtained from the stomach removed by gastrectomy after opening it along the greater curvature within one hour of resection. The tumour was classified according to Lauren [37].

### Extraction of bacterial DNA

Bacterium DNA obtained from 60 mm Petri dish growth was extracted using the QIAmp (QIAGEN, Hilden, Germany) kit according to manufacturer's recommendations with minor modifications. Distilled water was used as a reaction control. The DNA concentration was determined by spectrophotometry using NanoDrop 2000 (Thermo Scientific, Wilmington, NC) and stored at -20°C until use.

### Amplification of *H. pylori*-specific *ure*A and 16S *rRNA *genes

The presence of specific *ure*A and 16S *rRNA *genes was evaluated according to Clayton *et al. *[38] and Fox *et al*. [39] respectively. The standard Tx30a *H. pylori *strain was used as a positive control, and an *Escherichia coli *strain and distilled water were both used as negative controls.

The thermocycler GeneAmp PCR System 9700 (Applied Biosystems, Foster City, CA) was used for all reactions. The amplified products were electrophoresed in 2% agarose gel, stained with ethidium bromide, and analyzed in an ultraviolet light transilluminator.

### Amplification of the *cag*A gene

The *cag*A gene was amplified by means of two previously described primer pairs [40,41]. A *H. pylori *strain from our collection (1010-95), known to be *cag*A-positive, was used as a positive control, and Tx30a *H. pylori *strain lacking *cag*A and distilled water were both used as negative controls. The *H. pylori *strains were considered to be *cag*A-positive when at least one of the two reactions was positive.

### Amplification of the 3' variable region of *cag*A

For the PCR amplification of the 3' variable region of the *cag*A gene (that contains the EPIYA sequences), 20 to 100 ng of DNA were added to 1% Taq DNA polymerase buffer solution (KCl 50 mM and Tris-HCl 10 mM), 1.5 mM MgCl_2_, 100 μM of each deoxynucleotide, 1.0 U Platinum Taq DNA polymerase (Invitrogen, São Paulo, Brazil), and 10 pmol of each primer, for a total solution volume of 20 μL. The primers used were previously described by Yamaoka *et al*. [27]. The reaction conditions were: 95°C for 5 minutes, followed by 35 cycles of 95°C for 1 minute, 50°C for 1 minute, and 72°C for 1 minute, ending with 72°C for 7 minutes. The amplified products were electrophoresed in 1.5% agarose gel that was stained with ethidium bromide, and analyzed in an ultraviolet light transilluminator. The reaction yielded products of 500 to 850 bp according to the number of EPIYA C. This methodology also allows the detection of mixed infection.

### Sequencing of the 3' variable region of *cag*A

A significant subset of samples (around 75 patients of each group) was randomly selected for sequencing, in order to confirm the PCR results. PCR products were purified with the Wizard SV Gel and PCR Clean-Up System (Promega, Madison, MI) according to the manufacturer's recommendations. Purified products were sequenced using a BigDye^® ^Terminator v3.1 Cycle Sequencing Kit in an ABI 3130 Genetic Analyzer (Applied Biosystems, Foster City, CA). The sequences obtained were aligned using the CAP3 Sequence Assembly Program (available from: http://pbil.univ-lyon1.fr/cap3.php). After alignment, nucleotide sequences were transformed into aminoacid sequences using the Blastx program (available from: http://blast.ncbi.nlm.nih.gov/Blast.cgi) and compared to sequences deposited into the GenBank (http://www.ncbi.nlm.nih.gov/Genbank/).

### Determination of the serum PGI levels

The serum concentrations of PGI were determined in the patients with gastritis and duodenal ulcer by a specific ELISA (Biohit, Helsinki, Finland) according to manufacturer's recommendations.

### Statistical analysis

A sample size of at least 112 subjects in each group, in order to show a 15% difference among groups with a power of 80%, alpha of 5%, and confidence interval of 95% was calculated with the Epi Info program version 3.5.1 (Centres for Disease Control and Prevention, Atlanta, GA).

Association between the number of EPIYA C motifs and gastric cancer was initially evaluated in univariate analysis, and variables with a p-value less than 0.2 were included in the final model of logistic regression, controlling for the influences of age and sex. We also evaluated the effect of the gender and age in the number of EPIYA C segments in a model with the number of EPIYA C being the dependent variable and the age, sex and *H. pylori*-associated diseases as independent covariables. The logistic model fitness was evaluated with the Hosmer-Lemeshow test. Because the PGI levels were not normally distributed the data log transformed and became normal. Associations were, thus, evaluated by Student's t test (mean ± standard deviation). Association among the number of EPIYA C segments and the degree of gastric inflammation, atrophy and intestinal metaplasia was done by the two-tailed Mann-Whitney Test. The level of significance was set at a p value ≤ 0.05.

## Authors' contributions

SAB performed DNA extraction, PCR and sequencing. GAR, DMMQ and SAB participate in the design of the study and wrote the manuscript. AMCR carried out pepsinogen I evaluation and reviewed the manuscript. IEBS contributed to manuscript writing. MMDAC performed histological analysis. RCO participated in the discussion of the study design. DMMQ supervised laboratory work and analyzed the data. All authors read and approved the final manuscript.
